# Data shadows: When data become tangible, material, and fragile

**DOI:** 10.1016/j.patter.2025.101206

**Published:** 2025-03-14

**Authors:** Paul Trauttmansdorff, Kim M. Hajek

**Affiliations:** 1 Chair for Philosophy and History of Science and Technology & Ethical Data Initiative, Technical University of Munich, Munich, Germany

## Main text

How can data—their compositions, movements, and journeys through human activities and nonhuman surroundings—be visualized? This is the central question explored in *Data Shadows*, a film collaboration by artist and codirector Jacob van der Beugel, philosopher Sabina Leonelli, and codirector Oliver Page. Celebrating its premiere on January 18, 2025, in Munich’s Rio Filmpalast cinema, the film captivated a broad audience with its imaginative portrayal of data as tangible, material, and fragile objects. *Data Shadows* offers a powerful deconstruction of the dominant conception of data as abstract yet immutable numbers that mainly inhabit the virtual world. The film raises questions about the relationships between data and nature, between data materiality and motion, and between data extraction, origins, and contexts.

**Figure 1:**
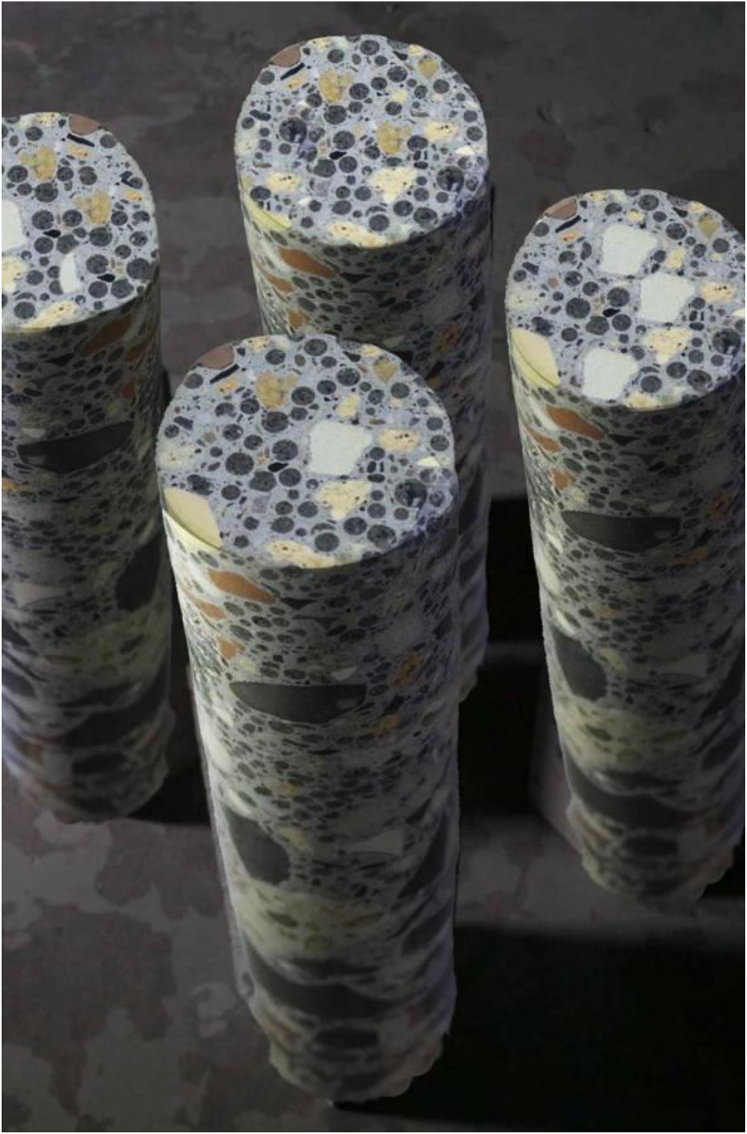
Excerpt from the film: The concrete cores Taken from A Philosophy of Open Science for Diverse Research Environments.

*Data Shadows* opens with close-up shots of a solid concrete cylinder, spinning, moving up and down, and taking shape within a circular container, a kind of repository in the ground. The cylinders, or “cores,” created by Jacob van der Beugel specially for the film (Figure 1), have notable solidity in the world. A single core might seem to represent one piece of data, yet each core is an aggregate of diverse materials, namely red, black, and ochre clumps visible in the gray concrete or dispersed through sedimented layers. In this way, they portray data as always an assemblage of information, as heterogeneous clusters formed through processes of production, extraction, and polishing. By repeatedly returning to these objects, the film effectively challenges our common perception of data as isolated digits, whether they are numerical, virtual, or homogeneous.

Throughout its sequences, *Data Shadows* construes data as a process, undergoing decontextualization from their material origins, histories, and environments. These sequences are accompanied by powerful, abstract, and consistent soundscapes created by composer Felix Erskine. Both visuals and sound evoke the “shadows” that persistently haunt data and that are beyond full control. As Leonelli states in a quote that also sets the tone of the film:

Data are material—whether in a digital or an analog form, whether made of bytes, soil, concrete, or wood, and whichever transformation they undergo as they journey across human landscapes—solid or liquid, corpuscular or massive.

**Figure 2:**
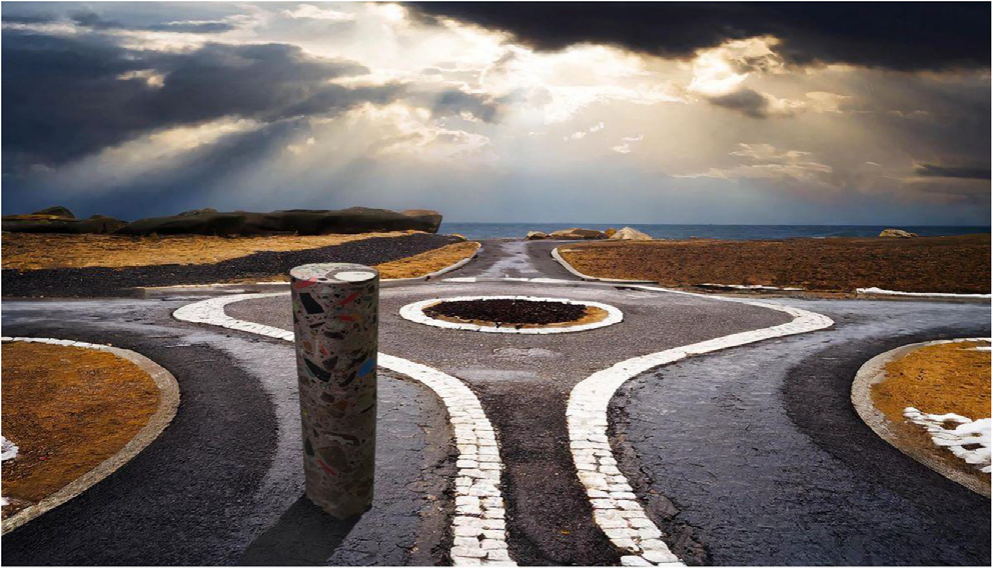
Excerpt from the film Taken from A Philosophy of Open Science for Diverse Research Environments.

The film is structured in three conceptual chapters. The first illustrates the labor-intensive processes of data extraction: how data are pulled from their broader surroundings and are continuously assembled and reassembled. Another chapter explores the diverse repositories and settings that shape and store data, symbolized through both mechanistic and natural landscapes. The third chapter focuses on data journeys, as three cores travel to different natural landscapes, where they undergo repeated layering, patterning, movement, and direction, often interacting with what the film suggests are the most foundational elements of life and the natural environment (Figure 2).

The film’s aesthetics revolve around two seemingly contrasting yet entangled themes. The first highlights colorful, somehow iconic elements of nature (water, light, air, and earth), emphasizing the diverse appearances of data, their constant motion, and their concrete, tangible forms. In one striking scene, a close-up captures vivid red liquid dripping into flowing water, coalescing, drifting away, and eventually disappearing. Are these red drops of lost data bleeding into oblivion? Should we mourn their loss? Or do they represent data that will no longer travel together, with the erosion of dripping water paralleling the ways that humans work on data and reconfigure their clusterings?

The second theme is darker, comprising mechanistic processes and operations that evoke the imagery of automated machines, industrial production, or even torture of “imprisoned” data. These sequences seem to represent the relentless processes that are done to and with data. Thus, one sequence shows how piercing beams of light do not so much illuminate a carefully positioned grid of cores as interrogate the shadows they create, drawing attention to the unseen. It was a fruitful surprise in the filmmaking process, Oliver Page commented after the screening, that some cores disappear from view under certain angles of illumination. These shadows reflect “data as part of a situation of inquiry—a context that shapes experiences, methods, technologies, answers to the questions humans ask of the world.” Data shadows hint at the often-hidden practices and infrastructures that bring data into visibility, pointing to that which is not seen, measured, or extracted, to what is missing or unavailable when making data present.^1^

Through both themes, we come to understand the vulnerability and fragility of data. While data may appear stable, they can just as easily be lost, destroyed, or corrupted. In a remarkable scene, the concrete core introduced at the beginning of the film is crushed and crumbled in slow motion, its materiality breaking apart. This moment illustrates that just as data are the result of material processes, their physicality can also vanish.

Another notable aspect of the film is the absence of humans. As the camera zooms in and out of nature, motion, and infrastructure, there is no direct depiction of human action. If this choice allows the film to focus on data as material entities, the invisibility of human involvement also points to the ways data have a life of their own; while humans may be part of their story, we cannot always control them.

The film also poses important ethical questions. In one of the final scenes, a prolonged bird’s-eye view reveals what appears to be an open-cast mine. This is a very explicit reminder of the immense resources required to produce and sustain data. While humans may not be visible in *Data Shadows*, their impact and traces on the Earth are unmistakably evident. The film effectively concludes with open questions: how can our work and interactions with data be guided by more sustainable values, situated within local environments and contexts? It invites us to look into the many stages of a data journey, from their creation in a lab, in the field, or in digital encounters, through their inscription in a repository to their interpretation and reuse by others. Where in these processes should we pay closer attention to the specifics of data production, sharing, and storage? What principles and values guide our decisions with data? And at which points in the data journey must we intervene, as humans, to foster more sustainable and resilient data practices?

These questions remained on the minds of the audience after the screening, as van der Beugel, Leonelli, and Page discussed the importance of demystifying data as immutable objects in an engaging panel discussion and conversation with the audience. At issue were the concepts of shadows and transparency that surround data as well as the dynamics of their visibility and invisibility. With many viewers impressed by the striking beauty of the film sequences, discussion finally turned to the role of aesthetics in data representations and collections and how these visual choices often shape our perception of what is considered “right” or “elegant” about data.

*Data Shadows* leaves viewers with a profound reflection on the materiality, fragility, and ethics of data, challenging us to reconsider their presence, movement, and impact on our shared planet.

The film will be made openly accessible in 2026. More information and clips about the film can be found at the Philosophy of Open Science project website.

## Declaration of interests

The authors declare no competing interests.
