# Data shadows: When data become tangible, material, and fragile

**DOI:** 10.1016/j.patter.2025.101230

**Published:** 2025-03-27

**Authors:** Paul Trauttmansdorff, Kim M. Hajek

## Main text

(Patterns *6*, 101206; March 14, 2025)

In the originally published version of this article, the authors misattributed the composer for the film *Data Shadows* as Felix Erskine. In fact, the soundscapes were created by composer Lucy Page, and this attribution has been corrected online. The authors apologize to the readers and to Lucy Page for the error.

